# Serum Short-Chain Fatty Acids in Colorectal Cancer: Diagnostic Performance and Decoupling from Gut Producer Abundance

**DOI:** 10.3390/cells15121096

**Published:** 2026-06-16

**Authors:** Juan Vicente-Valor, Sofía Tesolato, María Paz Lorenzo, Sofía de la Serna, Inmaculada Domínguez-Serrano, Jana Dziakova, Daniel Rivera, Francisco-Javier Rupérez, Antonio Torres, Antonia García, Pilar Iniesta

**Affiliations:** 1 Department of Biochemistry and Molecular Biology, Faculty of Pharmacy, Complutense University, Pza Ramón y Cajal s/n (Ciudad Universitaria), 28040 Madrid, Spain; juavicen@ucm.es (J.V.-V.); sofiteso@ucm.es (S.T.); 2 San Carlos Health Research Institute (IdISSC), San Carlos Hospital, 28040 Madrid, Spain; sofiacristinadela.serna@salud.madrid.org (S.d.l.S.); inmaculadadominguezserrano@gmail.com (I.D.-S.); jana.dziakova@salud.madrid.org (J.D.); daniel.rivera@salud.madrid.org (D.R.); antoniojose.torres@salud.madrid.org (A.T.); 3 Center for Metabolomics and Bioanalysis (CEMBIO), Faculty of Pharmacy, San Pablo-CEU University, CEU Universities, Monteprincipe Urbanization, Boadilla del Monte, 28660 Madrid, Spain; pazloga@ceu.es (M.P.L.); ruperez@ceu.es (F.-J.R.); antogar@ceu.es (A.G.); 4 General and Digestive System Surgery Service, San Carlos Hospital, 28040 Madrid, Spain; 5 Department of Surgery, Faculty of Medicine, Complutense University, 28040 Madrid, Spain

**Keywords:** short-chain fatty acids, colorectal cancer, biomarkers

## Abstract

Gut microbiota-derived short-chain fatty acids (SCFAs) shape epithelial and immune homeostasis, yet systemic SCFA profiles may diverge from gut microbial composition due to absorption and host metabolism. We quantified fasting serum SCFAs in 36 surgically resected colorectal cancer (CRC) patients and 20 cancer-free controls using targeted high-performance liquid chromatography–triple quadrupole mass spectrometry, and integrated these data with fecal and serum bacterial DNA profiles generated by 16S ribosomal RNA sequencing and functional inference. CRC was associated with a distinct circulating SCFA pattern: total SCFAs and acetate were increased, branched SCFAs were higher, and butyrate and valerate were lower relative to controls. Despite this clear systemic signature, associations between serum SCFAs and the relative abundance (RA) of putative SCFA-producing genera were sparse and inconsistent across CRC and control groups, both when considering fecal producers and serum-detected taxa. Interestingly, the total RA of SCFA-producing genera was higher in controls in feces but higher in CRC in serum, further supporting compartment-specific decoupling. Finally, several circulating SCFAs showed inverse associations with indicators of tumor progression within CRC. These results motivate integrative microbiota–metabolite studies and validation in larger cohorts to clarify how circulating SCFAs relate to gastrointestinal disease biology and immune regulation.

## 1. Introduction

Colorectal cancer (CRC) remains one of the most frequently diagnosed malignancies and a leading cause of cancer-related mortality worldwide. Despite significant advances in screening, surgical techniques, and systemic therapies, CRC incidence continues to rise in the global population [1]. This trend highlights the need to identify novel diagnostic or prognostic biomarkers and to unravel new molecular mechanisms in this disease.

Increasing evidence indicates that CRC development and progression are not solely driven by genetic and epigenetic alterations within tumor cells [2], but are profoundly influenced by environmental factors, host immunity, and the intestinal microenvironment. Among these, the gut microbiota and its metabolic products have emerged as critical regulators of intestinal homeostasis and colorectal carcinogenesis [3].

Short-chain fatty acids (SCFAs), primarily acetate, propionate, and butyrate, are key microbial-derived metabolites produced through the fermentation of non-digestible dietary fibers by anaerobic bacteria in the colon. A fraction of SCFAs named branched short-chain fatty acids (BSCFAs), such as isobutyrate, isovalerate and 2-methylbutyrate, come from the degradation of amino acids, particularly valine, leucine and isoleucine, and their role in human health and disease remains less studied. Given the colon’s direct exposure to high concentrations of SCFAs [4], alterations in their production or availability may have profound implications for colorectal epithelial biology and tumorigenesis. Consequently, research on SCFAs has largely focused on their measurement in feces. In contrast, relatively few studies have examined their concentrations in serum, likely due to the need for sensitive quantification methods, as their concentration is lower in serum than in feces. However, considering that gut absorption and systemic first-pass metabolism can substantially alter circulating SCFA levels, often resulting in poor correlations with fecal concentrations [5,6], the potential “endocrine” effects of SCFAs may be inaccurately inferred when systemic levels are not assessed.

Both straight SCFAs and BSCFAs play a central role in maintaining intestinal barrier integrity, regulating inflammation, modulating host metabolism, and shaping immune responses [7,8]. Therefore, alterations in their concentrations may contribute to a metabolic milieu that either restrains or, less frequently, promotes colorectal tumorigenesis [9]. Some SCFAs, especially butyrate [10], can serve as an energy source in colonic epithelial cells and support epithelial differentiation and barrier function. Additionally, both BSCFAs and other SCFAs act through epigenetic regulation via histone deacetylase (HDAC) inhibition and signaling primarily via G-protein-coupled receptors such as GPR41, GPR43, and GPR109A [11]. These pathways lead to the modulation of the expression of effector and immune checkpoint molecules [12], and the execution of anti-proliferative and pro-apoptotic effects [13]. Controversially, some SCFAs have also shown to exert a pro-tumorigenic role in certain contexts. Acetate uptaken by non-small-cell lung cancer cells, beyond acting as a main carbon source, induces oncoprotein c-Myc acetylation, thereby promoting tumor growth and immune evasion through programmed death-ligand 1 (PD-L1) expression [14]. The role of BSCFAs in cancer is still underexplored, although their levels have been suggested as a marker of colonic protein fermentation, which leads to several byproducts like ammonia or p-cresol with a known effect on DNA damage or colon carcinogenesis [15].

These seemingly contradictory findings underscore the need for a deeper understanding of SCFA biology in human cancer, and to untangle the influence of concentration, tumor stage, metabolic state of the cells and the surrounding immune microenvironment. Additionally, understanding how SCFA concentrations differ between CRC patients and cancer-free individuals is essential to elucidate their role in disease initiation and progression. This knowledge may guide the manipulation of the gut microbiota and its metabolites through dietary interventions, probiotics, and microbiota-targeted therapies. In this context, the present study aims to investigate the diagnostic impact of SCFAs in surgically resected CRC patients. By characterizing serum SCFA profiles in these populations and comparing them to profiles in cancer-free individuals, we seek to contribute to a more comprehensive understanding of the relationship between microbial metabolism and colorectal carcinogenesis, which may inform future strategies for cancer prevention and risk stratification. Results from our study support a distinctive signature of circulating SCFAs in patients with surgically resected CRC, which shows a relationship with colorectal tumor progression.

## 2. Materials and Methods

### 2.1. Patients and Samples

The study included 36 CRC patients who had undergone curative intent surgery at the San Carlos Hospital (HCSC, Madrid, Spain) between 2021 and 2023. Twenty cancer-free subjects were also recruited at the same hospital and included as the control group. Both subject populations came from two cohorts previously included in a publication from our team [16], where the results of fecal microbiota analysis were presented and the data were used to develop predictive cancer microbial signatures. Both cohorts were also included in a recent publication regarding the potential of other serum metabolites and of circulating bacterial DNA for CRC detection [17]. Data related to bacterial taxonomy in feces and serum samples were thus included in the publications mentioned above and have been reanalyzed for this study regarding SCFA production.

Exclusion criteria during recruitment were previous history of inflammatory bowel disease or gastrointestinal resection surgery, and antibiotic treatment in the month prior to obtaining the stool sample. For CRC patients, chemo- and/or radiotherapy prior to surgery were also considered as exclusion criteria. Regarding the control group, subjects with family history of cancer or personal oncological history, regardless of the time elapsed, were not included. Written approval to develop this study was obtained from the Clinical Research Ethics Committee of the HCSC (C.I. 19/549-E_BC, 27 December 2019). In addition, a signed written informed consent was retrieved from all participants prior to investigation.

The clinicopathological characteristics of the study cohorts are depicted in Table 1. Subjects were recruited subsequently regardless of age, gender or cancer tumor–node–metastasis (TNM) stage. Variables collected included age, gender, body mass index (BMI), prevalence of the main comorbidities and tumor-related variables for CRC patients. Based on their BMI values, all subjects were categorized as normal weight, overweight or obese according to the World Health Organization (WHO) criteria. CRC cases were staged following the American Joint Committee on Cancer classification [18].

Both feces and serum samples were obtained from all cases. Fresh fecal samples were collected before surgery or any systemic treatment, and frozen at −80 °C in tubes containing a DNA stabilizer (*Real^®^ stool sample collection microbiome kit*, Durviz, Valencia, Spain). Blood was also collected prior to surgery after an overnight fast and centrifuged for 10 min at 1300× *g* for serum separation. After collection, serum samples were stored in aliquots at −80 °C.

### 2.2. DNA Extraction and Bacterial 16S Ribosomal RNA (rRNA) Amplicon Sequencing

DNA extraction was performed on fecal samples using the *QIAamp^®^ Fast DNA Stool Mini Kit* (Qiagen, Hilden, Germany), and from serum samples with the *QIAamp^®^ DNA MiniKit* (Qiagen, Hilden, Germany). Subsequently, DNA was quantified using the Invitrogen™ Qubit™ 3 Fluorometer with the double-stranded DNA (dsDNA) High Sensitivity (HS) Assay (Thermo Fisher Scientific, Waltham, MA, USA). Microbiota analysis consisted of the amplification and sequencing of the bacterial 16S rRNA gene using Ion Torrent™ technology (Thermo Fisher Scientific, Waltham, MA, USA). The method for library preparation and sequencing has been described previously for fecal and serum samples [16,17].

### 2.3. Targeted SCFA Analysis Through High-Performance Liquid Chromatography–Triple Quadrupole Mass Spectrometry (HPLC-QqQ/MS)

SCFAs in serum samples were analyzed using a targeted metabolomics approach by high-performance liquid chromatography–triple quadrupole mass spectrometry. The study was performed following our previously validated protocol [19]. Briefly, 100 µL of each serum sample was mixed with 20 µL of an internal standard (IS) mixture containing labeled SCFAs (700:700:600, H_2_O:IS working solution:acetonitrile or ACN, *v*/*v*/*v*) and subsequently transferred to Centrifree Millipore^®^ (30 kDa) filters (Merck KGaA, Darmstadt, Germany) for protein removal by centrifugation. Standard solutions for each SCFA were also prepared by mixing 100 µL of the corresponding standard with 20 µL of the IS mixture. The resulting sample extracts or standard mixtures were subjected to derivatization by adding N-(3-dimethylaminopropyl)-N′-ethylcarbodiimide (EDC), 1-hydroxy-7-azabenzotriazole (HOAT) solution (10 mM, H_2_O), and then dansylhydrazine (Dns-Hz) solution (10 mg/mL, ACN). CuCl_2_ solution (100 mM, H_2_O) was subsequently used to quench the reactions. The analysis was carried out in an Agilent 1260 Infinity HPLC system coupled to an electrospray ionization source (ESI) in the positive mode in an Agilent G6470A QqQ/MS (Agilent Technologies, Santa Clara, CA, USA). Twenty µL of each derivatized sample or standard was injected into a reverse-phase column with a suitable precolumn (Acquity BEH C8 100 mm × 2.1 mm, 1.7 µm) from Waters (Mildford, MA, USA) thermostated in a UHPLC column oven (1290 Infinity II Multicolumn Thermostat, Agilent, Santa Clara, CA, USA) at 60 °C. The mobile phase consists of eluent A (5% ACN and 0.1% formic acid, FA, in ultrapure water) and eluent B (0.1% FA in ACN). They were pumped at a 0.350 mL/min flow rate with a total run time of 19.7 min. The method gradient started at 30% of B and went up to 45% in 8.5 min and it was held until 11.5 min. Then it went up to 90% at 11.6 min and was held until 13.6 min. Lastly, the initial conditions were restored in 0.1 min and the system was re-equilibrated for 6 min until the end of the analysis. Vials were set in a multisampler at 4 °C. Data were collected via dynamic multiple reaction monitoring (MRM), following previously studied transitions for these compounds (Table 2). The capillary voltage was set at 3500 V, the gas temperature was 200 °C, and the flow was 7 L/min. The nebulizer was fixed at 35 psi and the sheath gas temperature and flow rate were 250 °C and 7 L/min, respectively.

Quantification was based on the internal standard calibration with stable isotope standards *(Area Acid/Area labeled Acid)* considering the same counterpart acids, except for isobutyric, for which either D3-butyric acid or butyric acid-1-^13^C was used, and for 2-methylbutyric and isovaleric for which d9-valeric acid or butyric acid-1-^13^C was used, as per the internal reference standard according to the closest standard in retention time. Data were collected in dynamic multiple reaction monitoring (MRM).

### 2.4. Bioinformatics and Statistical Analyses

The fecal and serum bacterial DNA data used in this study came from two previous publications from our group [16,17], as mentioned above, and had been subjected to bioinformatics analyses using the Quantitative Insight Into Microbial Ecology 2 (QIIME2) pipeline [20]. The obtained Operational Taxonomic Units (OTUs) were assigned to taxonomic categories ranging from bacterial phylum to bacterial genus, and relative abundance (RA, %) values of the resulting bacterial taxa were calculated as the proportion of OTUs of each taxon with respect to the total features of the sample. Predictive functional profiles of the fecal microbiota were also inferred using the Phylogenetic Investigation of Communities by Reconstruction of Unobserved States (PICRUSt2) v2.5.0 tool [21].

Statistical analyses were conducted using STATA IC16.1 (Stata-Corp LLC, College Station, TX, USA). Both SCFA concentrations and bacterial RA were compared between groups using the Mann–Whitney U test (two groups) or the Kruskal–Wallis test (three or more groups). The diagnostic capability of serum SCFAs was further evaluated by receiver operating characteristic (ROC) curves to optimize the area under the curve (AUC) [22]. The optimal discrimination threshold was determined using the Euclidean distance method implemented in the Cutoff Finder application [23]. Logistic regression analysis was performed to adjust for potential confounders between populations. Spearman rank correlations were performed to assess associations between SCFA concentrations and either bacterial producers or clinicopathological variables. Differences in SCFA concentrations across categories of anatomopathological variables were also evaluated using both continuous and categorized SCFA values. In this case, categorization of SCFA concentration was determined using the log-rank test implemented in the Cutoff Finder application. In all cases, a *p* value < 0.05 was considered as statistically significant.

## 3. Results

### 3.1. Comparison of Serum SCFA Concentration Between CRC Patients and Controls

**Figure 1:**
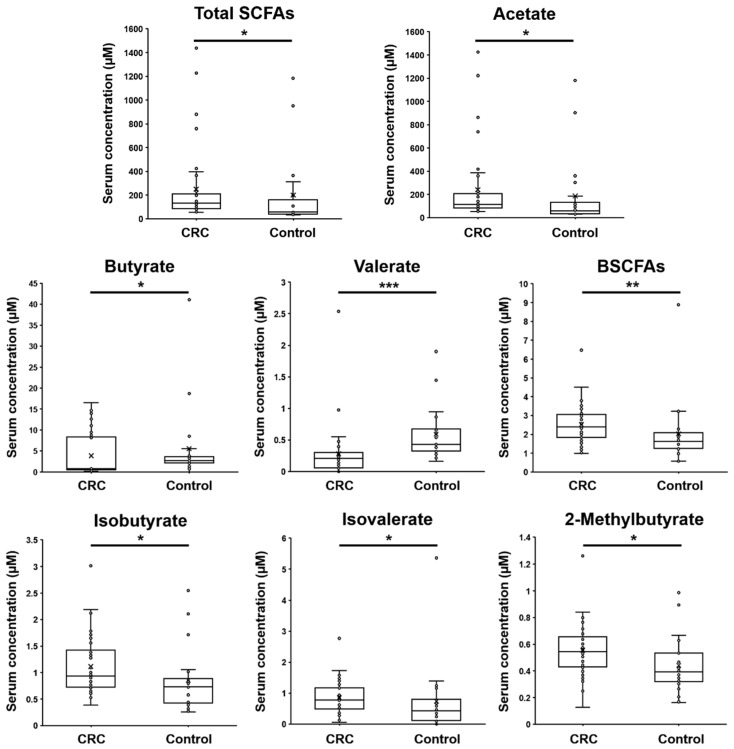
Boxplots showing serum SCFAs whose concentration was significantly different between CRC patients and the control group. Total SCFAs: sum of acetate, propionate, butyrate, valerate, isobutyrate, isovalerate and 2-methylbutyrate; BSCFAs: sum of isobutyrate, isovalerate and 2-methylbutyrate. * *p* < 0.05, ** *p* < 0.01, *** *p* < 0.001 (Mann–Whitney U test).

First, circulating levels of the analyzed SCFAs were compared between the CRC patients and the control cancer-free subjects (Figure 1 and Table 3). The CRC group showed significantly higher serum concentration of total SCFAs (*p* = 0.010) and of acetate (*p* = 0.010). The same tendency was found for total BSCFA levels (*p* = 0.008), as well as in the concentration of individual isobutyrate (*p* = 0.034), isovalerate (*p* = 0.026), and 2-methylbutyrate (*p* = 0.022). On the contrary, CRC patients had significantly decreased serum concentrations of both butyrate (*p* = 0.017) and valerate (*p* < 0.001) with respect to cancer-free individuals. No significant differences were found in the serum levels of propionate (*p* = 0.166).

**Figure 2:**
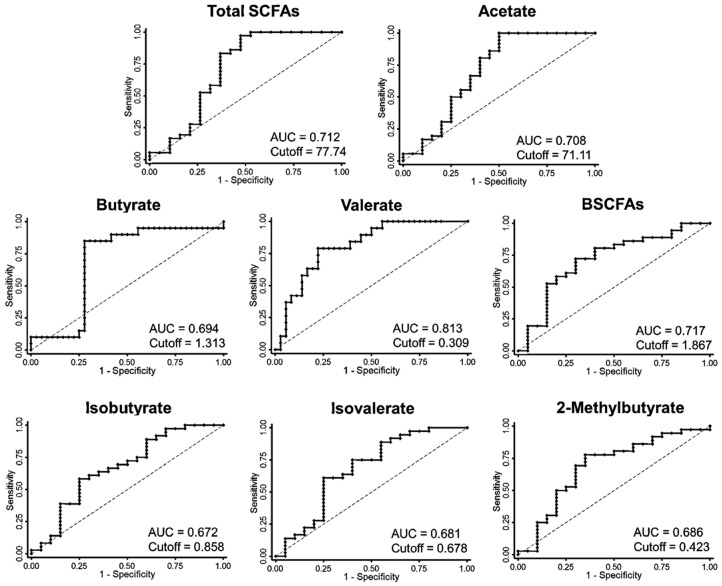
Receiver operating characteristic (ROC) curves showing the independent diagnostic accuracy, on our study population, of the serum concentration (in µM) of the differential SCFAs. AUC: area under the curve; Total SCFAs: sum of acetate, propionate, butyrate, valerate, isobutyrate, isovalerate and 2-methylbutyrate; BSCFAs: sum of isobutyrate, isovalerate and 2-methylbutyrate.

The diagnostic capability of the differential serum SCFAs was assessed by obtaining ROC curves (Figure 2). For the SCFAs differentially increased in CRC sera, the event condition considered was the presence of this cancer, whereas for the SCFAs increased in the control group, the event condition was the absence of cancer. The optimal cutoff point was calculated through the Cutoff Finder application using the Euclidean algorithm.

Serum valerate displayed the best diagnostic accuracy, with an AUC value of 0.813. The diagnostic performance of serum total SCFAs, acetate or total BSCFAs was lower (AUC value of 0.71), whereas butyrate, isobutyrate, isovalerate and 2-methylbutyrate obtained AUC values below 0.70.

**Figure 3:**
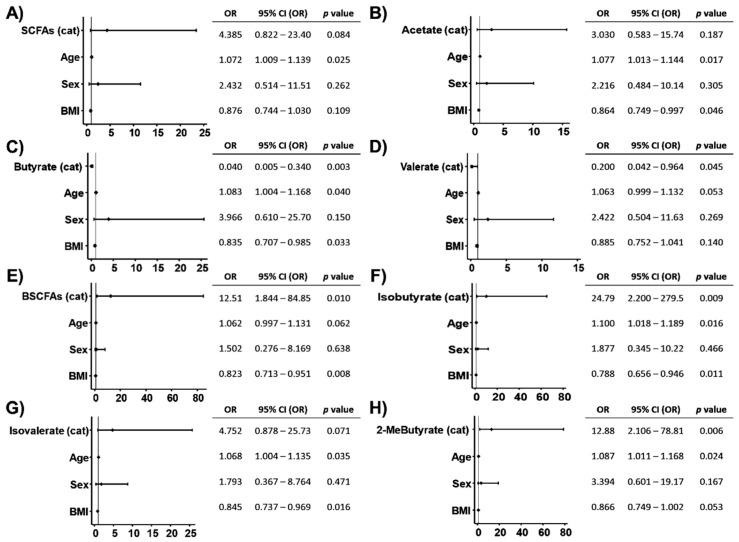
Forest plots and results from the multivariate logistic regressions, regarding the predictive capability for CRC of the studied SCFAs after adjusting for age, gender and BMI value. (**A**) Total SCFAs: acetate, propionate, butyrate, valerate, isobutyrate, isovalerate and 2-methylbutyrate (cutoff: 77.74). (**B**) Acetate (cutoff: 71.11). (**C**) Butyrate (cutoff: 1.313). (**D**) Valerate (cutoff: 0.309). (**E**) Total BSCFAs: isobutyrate, isovalerate and 2-methylbutyrate (cutoff: 1.867). (**F**) Isobutyrate (cutoff: 0.858). (**G**) Isovalerate (cutoff: 0.678). (**H**) 2-Methylbutyrate (cutoff: 0.423). Cat: categorized; CI: confidence interval; OR: odds ratio.

The CRC and control populations had relevant dissimilarities in clinicopathological features that could be acting as confounders in the differences found for serum SCFAs. Particularly, age was significantly higher in CRC patients with respect to controls (*p* < 0.001). The distribution of genders and BMI groups was also significantly different in both groups (*p* = 0.026 and *p* = 0.003, respectively). Thus, differential SCFAs were adjusted by age, gender and BMI through multivariate logistic regression. Previously, the concentration of each SCFA had been dichotomized according to the threshold calculated through the Cutoff Finder application. Results from the multivariate analyses, and the associated forest plots, are shown in Figure 3.

As observed, circulating butyrate and valerate levels acted as protective variables after adjusting for confounders (*p* = 0.003 and *p* = 0.045, respectively). On the contrary, total BCFAs and individual isobutyrate and 2-methylbutyrate maintained a significant association with CRC risk independently from age, gender, and BMI (*p* = 0.010, *p* = 0.009 and *p* = 0.006, respectively). An association bordering statistical significance was found between CRC and either serum total SCFAs (*p* = 0.084) or isovalerate (*p* = 0.071).

Due to limited sample size, which could preclude robust conclusions on the role of diagnostic performance of SCFAs, we performed additional sensitivity analyses including Firth logistic regression, bootstrap resampling and stratified cross-validation. These analyses showed directionally consistent associations for butyrate, BSCFAs, isobutyrate, and 2-methylbutyrate with CRC status, after adjustment for sex, age, and BMI (Table 4).

Overall, these results suggest that the observed associations are not driven by sparse-data bias; however, predictive performance should be interpreted with caution given the limited sample size and variability in cross-validated estimates.

### 3.2. Association Between Circulating SCFA Levels and the Abundance of Bacterial Producers in Feces and Serum

Based on the aforementioned data from the fecal microbiota and serum bacterial DNA analyses performed on CRC patients and controls [16,17], we explored the connections between serum SCFAs and the fecal and serum RA of known SCFA-producing bacteria. A list of the SCFA producers investigated can be found in the Supplementary Materials (Table S1), along with the supporting literature for their role as producers of these metabolites [24,25,26,27,28,29,30,31,32,33,34,35,36,37].

**Figure 4:**
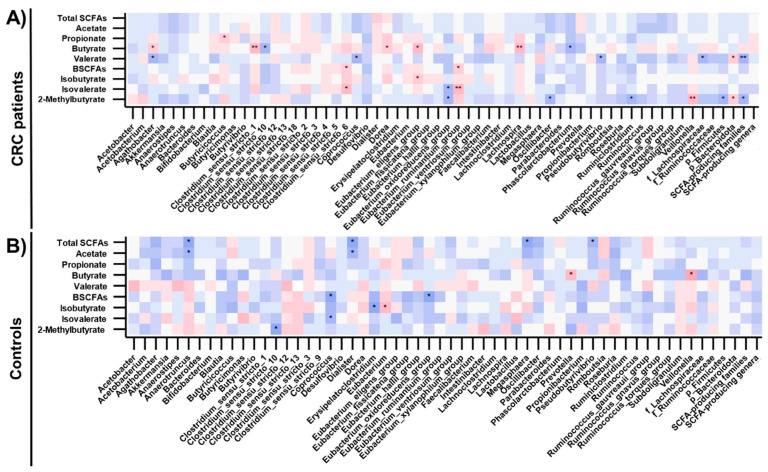
Correlation heatmaps between the fecal relative abundance (RA) of SCFA-producing gut bacterial genera and serum SCFA concentration. Heatmaps were retrieved for (**A**) the CRC group and (**B**) the control group. * *p* < 0.05, ** *p* < 0.01. Blue colors indicate negative correlations, and red colors indicate positive correlations.

Regarding the relationship with fecal microbiota (Figure 4), some significant positive correlations were detected between serum SCFA concentrations and the RA of SCFA-producing bacteria. Particularly, in CRC patients, serum propionate was correlated to fecal RA of genus *Butyricicoccus* (correlation coefficient or r = 0.388, *p* = 0.019). Butyrate levels were correlated to the RA of genera *Agathobacter* (r = 0.361, *p* = 0.031), *Clostridium sensu stricto 1* (r = 0.445, *p* = 0.006), *Dorea* (r = 0.356, *p* = 0.033), *[Eubacterium] eligens group* (r = 0.415, *p* = 0.012) and *Lachnospira* (r = 0.427, *p* = 0.009) in the CRC group, and to the RA of genera *Prevotella* (r = 0.483, *p* = 0.031) and *Veillonella* (r = 0.558, *p* = 0.011) in the control group. Moreover, a correlation was found in CRC patients between the total fecal RA of phylum Bacteroidota and the serum levels of valerate (r = 0.364, *p* = 0.029). Regarding BSCFAs, circulating isobutyrate was correlated with the fecal RA of genus *[Eubacterium] eligens group* (r = 0.377, *p* = 0.024) in CRC patients, and of genus *Eubacterium* (r = 0.463, *p* = 0.040) in controls. Both total BSCFAs and isovalerate levels in CRC serum samples were correlated to fecal *Clostridium sensu stricto 6* (r = 0.394 and *p* = 0.017 for total BSCFAs; r = 0.423 and *p* = 0.010 for isovalerate) and *[Eubacterium] ruminantium group* (r = 0.361 and *p* = 0.031 for total BSCFAs; r = 0.473, *p* = 0.004 for isovalerate). Also in CRC cases, correlations were found between serum concentration of 2-methylbutyrate and fecal RA of both phylum Bacteroidota (r = 0.364, *p* = 0.029) and genus *Veillonella* (r = 0.452, *p* = 0.006).

Despite the individual associations mentioned, correlations between serum SCFAs and fecal bacterial producers were scarce overall. Of 1071 correlations, only 1.6% were positive and significant, and depended on the population group considered. Moreover, correlations were generally not consistent between the two groups.

**Figure 5:**
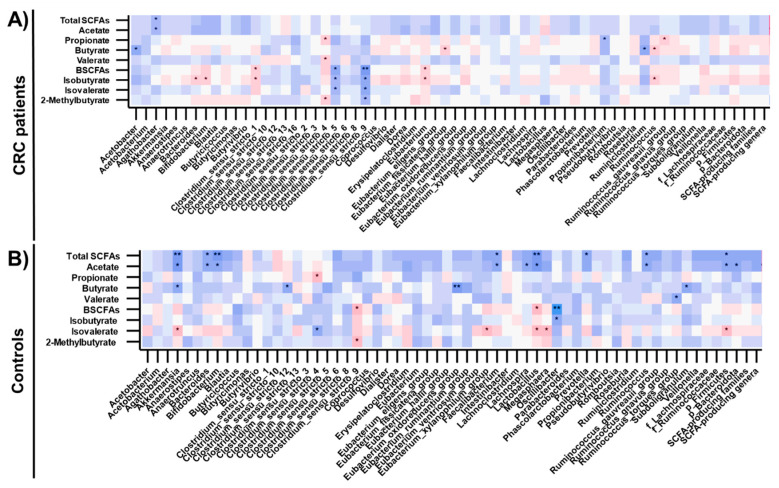
Correlation heatmaps between the serum RA of SCFA-producing bacterial genera and serum SCFA concentration. Heatmaps were retrieved for (**A**) the CRC group and (**B**) the control group. The positive and significant correlations with individual SCFA are indicated below each panel. * *p* < 0.05, ** *p* < 0.01. Blue colors indicate negative correlations, and red colors indicate positive correlations.

Correlations were also explored between SCFA-producing bacteria detected in serum samples and serum SCFA concentrations (Figure 5). Focusing on the positive associations, some significant correlations were found in CRC cases involving the levels of propionate, butyrate and valerate. Both circulating propionate and valerate were correlated with the RA of genus *Clostridium sensu stricto 4* (r = 0.368 and *p* = 0.032 for propionate; r = 0.342 and *p* = 0.048 for valerate). Serum propionate was also correlated to the RA of genus *[Ruminococcus] gauvreauii group* (r = 0.340, *p* = 0.049), and circulating butyrate with the RA of genera *[Eubacterium] fissicatena group* (r = 0.390, *p* = 0.023) and *Ruminococcus* (r = 0.348, *p* = 0.044). No relevant positive associations for these SCFAs were observed in the control group, except the correlation between serum propionate and the RA of *Clostridium sensu stricto 4* (r = 0.475 and *p* = 0.034). As for BSCFAs, serum isobutyrate in CRC patients correlated with the RA of genera *Bacteroides* (r = 0.343, *p* = 0.047), *Bifidobacterium* (r = 0.393, *p* = 0.022), *Clostridium sensu stricto 1* (r = 0.379, *p* = 0.027), *Eubacterium* (r = 0.361, *p* = 0.036) and *Ruminococcus* (r = 0.357, *p* = 0.038). Genera *Clostridium sensu stricto 1* and *Eubacterium* were also correlated to the serum total BSCFAs in the CRC group (r = 0.399 and *p* = 0.019 for *Clostridium sensu stricto 1*; r = 0.363 and *p* = 0.035 for *Eubacterium*). In controls, serum isovalerate was correlated to the RA of genera *Akkermansia* (r = 0.523, *p* = 0.018), *[Eubacterium] xylanophilum group* (r = 0.488, *p* = 0.029), *Lactobacillus* (r = 0.515, *p* = 0.020) and *Megasphaera* (r = 0.454, *p* = 0.044), and to phylum Firmicutes (r = 0.475, *p* = 0.034). A positive correlation was also found in controls between serum total BSCFA levels and the RA of *Clostridium sensu stricto 9* (r = 0.510, *p* = 0.022) and *Lactobacillus* (r = 0.459, *p* = 0.042). Finally, levels of circulating 2-methylbutyrate were correlated with the RA of *Clostridium sensu stricto 4* (r = 0.366, *p* = 0.034) in CRC patients, and of *Clostridium sensu stricto 9* (r = 0.459, *p* = 0.042) in controls. Similarly to what happened with fecal microbiota, only 1.94% of the 1134 correlations explored were positive and significant, and varied depending on the group considered.

**Figure 6:**
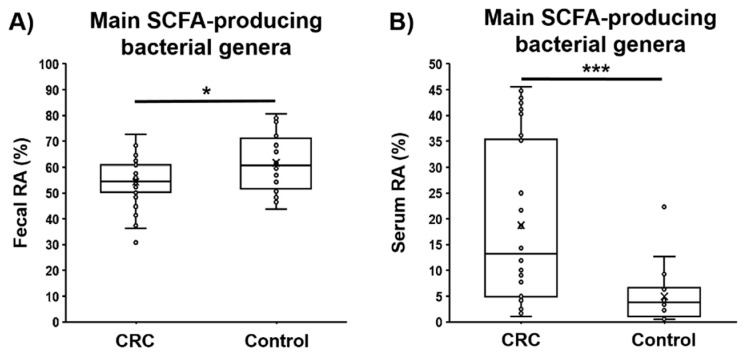
Boxplots showing the comparison of total RA of the main SCFA-producing gut bacterial genera between CRC patients and controls in (**A**) fecal samples and (**B**) serum samples. * *p* < 0.05, *** *p* < 0.001.

We compared the total RA of SCFA-producing bacterial genera between CRC patients and controls. In feces, values were significantly higher in the control group (Figure 6A, median RA [interquartile range or IQR] of 54.51 [50.43–60.91] in CRC patients vs. 60.64 [51.67–70.18] in controls, *p* = 0.049). On the contrary, total serum RA of SCFA producers was significantly higher in CRC patients (Figure 6B, median RA [interquartile range or IQR] of 13.24 [4.95–35.16] in CRC patients vs. 3.79 [1.12–6.56] in controls, *p* < 0.001).

Finally, we explored the functional profile of gut microbiota from CRC patients and controls, inferred from the taxonomic data using PICRUStParticularly, Metacyc pathways potentially involved in the direct production of SCFAs were compared. The full list of SCFA-related pathways and their combinations considered is provided in the Supplementary Materials (Table S2).

**Figure 7:**
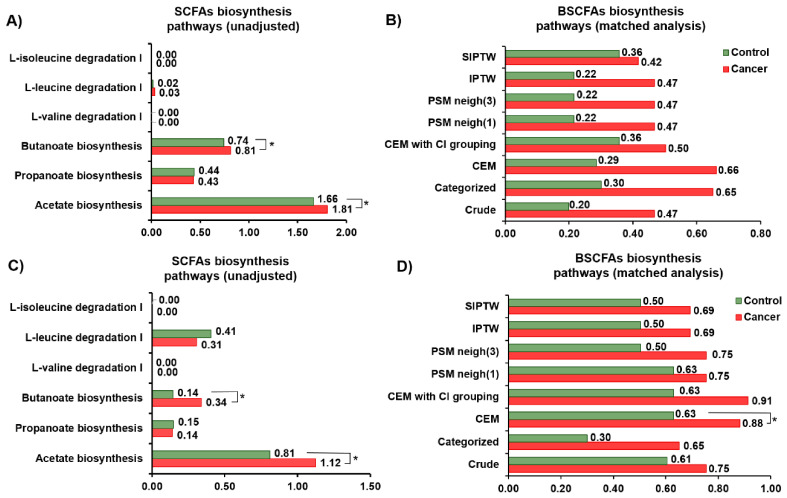
(**A**,**C**) Barplots showing the median predicted relative counts and comparison of the Metacyc pathways related to the biosynthesis of SCFAs in feces and in serum samples, respectively; (**B**,**D**) barplots showing the median relative predicted counts and comparison of the Metacyc valine, leucine and isoleucine degradation routes, linked to the biosynthesis of BSCFAs, through different matched analyses by age, gender and BMI. SIPTW: standardized inverse probability of weighting; IPTW: inverse probability of weighting; PSM (neigh 1): propensity score matching; CEM with CI grouping: coarsened exact matching with confidence interval grouping; Categorized: values dichotomized according to medians; Crude: using continuous values. * *p* < 0.05 (Mann–Whitney U test).

As shown in Figure 7, pathways linked to the biogenesis of butyrate and acetate had significantly higher counts in the CRC group, both in feces (Figure 7A) and in serum samples (Figure 7C). However, these differences had not been adjusted for potential confounders. When the comparisons were performed in a matched manner by age, gender and BMI, counts regarding the valine, leucine and isoleucine degradation routes, related to the biosynthesis of BSCFAs, were numerically superior in CRC vs. controls according to all the matching methods applied in both feces (Figure 7B) and serum samples (Figure 7D), although most of the differences found were not significant (*p* > 0.05).

### 3.3. Relationship Between Circulating SCFAs and Colorectal Tumor Characteristics

To assess the relationship between serum SCFAs and CRC characteristics, an exploratory correlation study was performed between the SCFA concentrations and cancer-related variables, including the primary tumor location (right colon, left colon or rectum), the TNM stage and its constituent descriptors (T or “tumor size”, N or “lymph node invasion” and M or “metastasis”). There was a correlation between the TNM stage and the summed concentration of SCFAs (r = −0.35, *p* = 0.039). When TNM descriptors were analyzed separately, correlations were found between lymph node invasion (N category) and the serum concentration of total SCFAs (r = −0.37, *p* = 0.028) and acetate (r = −0.36, *p* = 0.031), as well as between the presence of metastasis (M category) and the concentration of total BSCFAs (r = −0.37, *p* = 0.031) and individual isovalerate (r = −0.35, *p* = 0.037).

Based on the results of the correlation study, the concentrations of SCFAs were compared between CRC patients with different TNM stages (Table 5). Cases were grouped into earlier stages I to IIIA against more advanced stages IIIB to IV, due to the small sample sizes in the stage I group (five cases) and the stage IV group (two cases).

**Figure 8:**
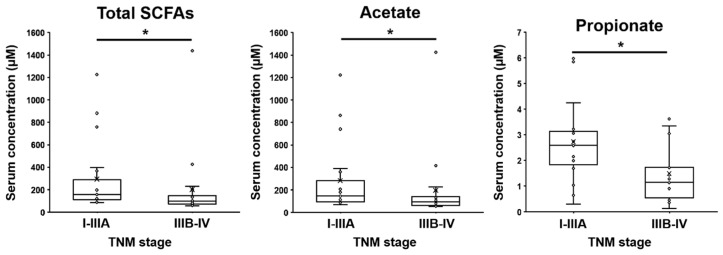
Boxplots showing serum SCFAs whose concentration was significantly different between CRC patients with different TNM stage. Total SCFAs: sum of acetate, propionate, butyrate, valerate, isobutyrate, isovalerate and 2-methylbutyrate. * *p* < 0.05 (Mann–Whitney U test).

As shown in the table, CRC cases from the stage IIIB–IV group had lower serum concentrations of several SCFAs compared to cases from the stage I–IIIA group. Differences were statistically significant for total SCFAs (*p* = 0.028), in line with the correlation results, as well as for acetate (*p* = 0.041) and propionate (*p* = 0.028), and borderline significant in the case of butyrate (*p* = 0.051), isobutyrate (*p* = 0.084), and isovalerate (*p* = 0.090) (Figure 8).

**Figure 9:**
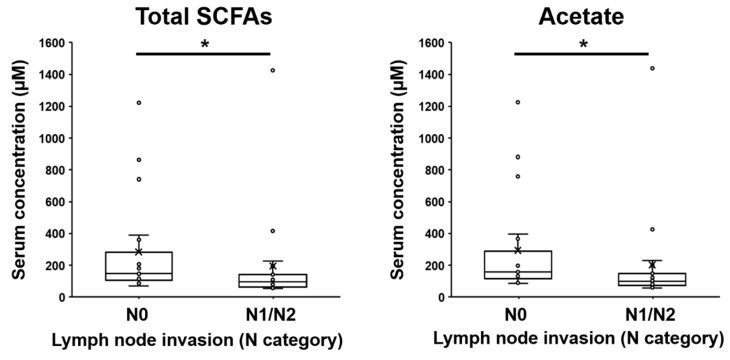
Boxplots showing the serum SCFAs whose concentration varied significantly between CRC cases with different degrees of lymph node invasion (N descriptor). Total SCFAs: sum of acetate, propionate, butyrate, valerate, isobutyrate, isovalerate, and 2-methylbutyrate. * *p* < 0.05 (Mann–Whitney U test).

We also compared the serum concentrations of SCFAs with respect to the individual N descriptor value (lymph node invasion) and M descriptor value (presence of metastasis) of CRC. For the comparison between CRC patients with different N categories (Table 6 and Figure 9), cases were grouped as N0 (without lymph node invasion) against N1 or N2 (with lymph node invasion) due to the reduced number of cases with N2 category (only four patients). CRC cases with lymph node invasion had significantly lower serum levels of both global SCFAs (*p* = 0.023) and acetate (*p* = 0.028), in line with the correlation results. No relevant differences were found in the concentrations of propionate (*p* = 0.274), butyrate (*p* = 0.350), valerate (*p* = 0.188), total BSCFAs (*p* = 0.456), isobutyrate (*p* = 0.496), isovalerate (*p* = 0.318) or 2-methylbutyrate (*p* = 0.812).

When SCFA concentrations were compared between CRC cases with or without distant metastasis (M descriptor), total circulating BSCFAs were significantly reduced in the metastatic group or M1 with respect to the group without metastasis or M0 (median value [range] of 1.15 [0.35] vs. 2.41 [5.45], *p* = 0.032 in Mann–Whitney U test). The same happened with circulating isovalerate levels (for M1 vs. M0, median value [range] of 0.22 [0.11] vs. 0.79 [2.72], *p* = 0.038 in Mann–Whitney U test). Isobutyrate was also diminished in serum from M1 cases, although differences only bordered statistical significance (for M1 vs. M0, median value [range] of 0.60 [0.15] vs. 0.97 [2.63], *p* = 0.062 in Mann–Whitney U test). These results are consistent with the negative correlations found between these SCFAs and the M category. However, they should be interpreted with caution because the M1 group only contained two cases.

## 4. Discussion

In this study, we investigated the diagnostic performance of serum levels of the SCFAs acetate, propionate, butyrate, valerate, and BSCFAs (isobutyrate, isovalerate and 2-methylbutyrate) in patients affected by CRC. We also confirmed that circulating SCFA levels cannot be inferred from the abundance of gut producers. Thus, we support the investigation of SCFA concentrations in serum, and not only in feces, as their systemic levels, which could exert distant effects, may not correlate with local intestinal ones. Furthermore, we provided a new comprehensive full list of gut SCFA-producing bacterial genera detectable by 16S metagenomics procedures, completing previously published lists [29,37], and related these genera to specific SCFAs. Finally, we defined a set of Metacyc pathways potentially associated with SCFA production and evaluated them in our population. To the best of our knowledge, SCFA-specific biosynthesis pathways have not yet been included as such in PICRUSt2 analyses.

Based on the positive effects of SCFAs, we could have expected an increase in serum levels of both straight SCFAs and BSCFAs in cancer-free controls with respect to CRC patients. However, results show that this preconceived idea was inaccurate and depends on the specific SCFA studied. Particularly, an increase in serum total SCFAs (straight and branched), acetate and the three BSCFAs was detected in CRC patients with respect to controls, while butyrate and valerate were increased in the control group. These associations were further confirmed by logistic regression analysis, which also showed an independence from confounders related to age, gender and BMI value except for total SCFAs, acetate and isovalerate.

The significant decrease detected for serum butyrate and valerate in CRC matches their mainly protective role against the development of this cancer [8]. Diminished fecal butyrate levels are generally observed in CRC, based on the results of a recent meta-analysis [9]. In contrast, fecal valerate has been reported as increased in patients with this cancer [38,39]. Research on circulating SCFAs in CRC is currently scarce, although a recent study indicated a decrease in plasma levels of both butyrate and valerate in CRC patients with respect to cancer-free individuals [40], which is consistent with our findings and highlights the discrepancies found between local and systemic SCFAs. In contrast to the results from the latter study indicating an increase in circulating propionate in CRC, we did not observe significant differences in the serum levels of this SCFA between the CRC and the control groups. Nevertheless, fecal propionate levels appear to be generally unaffected by CRC development [9].

Regarding acetate, the increase in serum concentration found in our CRC population is inconsistent with the general downward trend observed in feces [9], which suggests a main source other than microbial production affecting systemic levels of this SCFA. Acetate is involved in anaplerotic routes and can be synthesized in the liver [41]. Endogenous metabolism is thus an important factor contributing to its circulating levels, although other processes may also be participating. For example, global histone deacetylation has been reported in cancer cells as a way to regulate their pH in response to an acidic microenvironment. The process releases acetate anions, which are co-exported with protons via monocarboxylate transporters (MCTs), thus preventing further decrease in intracellular pH [42]. This could contribute to the increment in the levels of this fatty acid in CRC patients, as well as the increase in total serum SCFA levels found in CRC patients, as it was the most abundant SCFA within this group. Of note, other authors also determined the presence of higher circulating acetate in CRC samples [40]. Importantly, in our study, both the differences in total SCFAs and in acetate were apparently influenced by age, gender or BMI. These three features are proven to have an impact on gut microbiota and its functions [43]. Their effect on SCFA production is also described in the literature, with a general decline of fecal carbohydrate-derived SCFAs in elderly individuals [44] and conflicting results regarding BMI, with some evidence supporting an increased production of the main SCFAs in obesity, which may be associated with increased energy harvest [45] and other evidence linking this condition to decreased SCFA concentrations [46]. Gender-related differences in fecal SCFA profile have also been noted [47].

Elevated fecal levels of BSCFAs, particularly isovalerate and isobutyrate, have been reported in CRC, similarly to the increase in BSCFAs observed in our CRC serum samples. These increments are thought to reflect disease-associated changes rather than direct toxicity, as BSCFAs have not been shown to harm intestinal epithelial cells. Potential sources include increased epithelial cell turnover and altered microbial mucolytic activity. Similar elevations in bacterial BSCFA production have also been observed in inflammatory bowel disease [48]. Notably, 2-methylbutyrate concentration was higher in the plasma from CRC patients according to another study [40]. Changes in diet might be a factor affecting BSCFA production, but this may also be related to a physiological shift in the metabolism of microbiota with aging, from mainly saccharolytic toward proteolytic and putrefactive, as it has been previously reported [44,49]. This would explain why, after adjusting for confounders including age differences between CRC and controls, the association between serum isovalerate and CRC was no longer significant. In fact, previous research showed that molar concentrations of BSCFAs in feces were strongly correlated with age, whereas no relationship was found with BMI [15].

In summary, we have described an association between CRC and the levels of several circulating SCFAs, including BSCFAs. Although in most cases this association showed statistical independence from age and BMI, both features may still be affecting SCFA levels and are also related to carcinogenesis. It is plausible that SCFAs act as molecular mediators linking microbiota composition to CRC development and progression. However, the cross-sectional nature of our study does not allow us to determine whether the observed alterations in SCFA levels are causes or consequences of the disease process. Our findings therefore support their potential role as biomarkers of CRC rather than establishing a causal relationship. Nevertheless, substantial experimental evidence suggests that SCFAs can directly influence tumor biology through multiple mechanisms, including modulation of inflammation, cellular proliferation, apoptosis, and immune responses. At the same time, changes in SCFA concentrations may also reflect the metabolic and microbial alterations associated with the disease state.

Regarding correlations between circulating SCFAs and fecal bacterial producers, we observed few significant associations, with only *Veillonella* consistently identified in correlations across both CRC patients and controls in fecal samples. Although we did not stratify the correlation analysis by specific producers and individual SCFAs, the overall sparsity of correlations suggests a decoupling between systemic SCFA levels and the abundance of their producers in the gut, as has been previously reported by other authors within larger study cohorts [6]. This dissociation might be explained by the fact that the SCFAs absorbed in the large intestine (up to 95% of the total produced SCFAs) are mainly used by colonocytes, with only a minor fraction reaching portal circulation. Except for acetate, most of the portal SCFAs are further metabolized by hepatocytes, and only a small proportion reaches the systemic circulation [50]. Moreover, circulating SCFAs may vary depending on the host metabolism, as mentioned previously. Despite this, all genera showing significant correlations are recognized as major SCFA producers, most of them reported in a comprehensive review of SCFA-producing bacteria [29]. Given the limited correlations with fecal producers, we hypothesize that circulating SCFA levels may also be affected by production at other locations. While straight-chain SCFAs are primarily derived from the diet, BSCFAs could be produced elsewhere following amino acid degradation. Consistently, we found scarce positive correlations between SCFA-producing bacteria identified via serum bacterial DNA analysis and circulating SCFA levels, with only *Clostridium sensu stricto 4* genus shared between CRC and control groups. Overall, these findings indicate that serum SCFA measurements may offer information not captured through analysis of gut SCFA producers.

Notably, many of the SCFAs increased in serum from CRC patients with respect to cancer-free subjects showed an inverse relationship with cancer progression. Particularly, levels of total SCFAs and of acetate decreased significantly in cancers with lymph node invasion, whereas total BSCFAs and isovalerate did in metastatic cases. The negative relationship between acetate and CRC invasion suggests a rather suppressive role of this SCFA on cancer progression, possibly through mechanisms like the induction of oxidative stress and mitochondrial dysfunction, which ultimately leads to cell apoptosis [8]. However, this metabolite can perform a double-edged effect on tumors, inhibiting tumor progression or sustaining metabolic demands for cell proliferation depending on the particular cellular and environmental conditions [51,52]. This context-dependent role could explain the paradoxical relationship found between circulating acetate and either cancer development or its progression. Nevertheless, as mentioned previously, circulating levels of this SCFA might not reliably reflect its functions on the tumor, but mainly the metabolic ones, given its high turnover. It is also worth considering that the increased serum acetate found in CRC patients vs. controls could be affected by the differences in age, gender and BMI between both populations, rather than being only related to the cancer process.

The role of BSCFAs as protective against CRC progression and metastasis could be exerted through several mechanisms, with the most important being their boost of antitumor activity. Besides its direct inhibition of cancer cell survival, isobutyrate has been shown to increase the expression of Programmed cell death protein 1 (PD-1) and Interferon gamma (IFN-γ) in T cells and decrease the expression of the transcription factor Forkhead box P3 (FoxP3) as well as the percentage of regulatory T cells [53]. However, the relationship of this BSCFA with CRC metastasis is currently controversial, as its fecal levels increased with inulin intake which was also shown to inhibit CRC liver metastasis [54], but another study found it was increased in the plasma of CRC patients with distant organ metastasis, and that it promoted metastasis both in vitro and in vivo [55]. Data regarding isovalerate are also controversial, as it seems to enhance 5-hydroxytryptamine production by enteric serotonergic neurons, which interacts with colon cancer stem cells activating Wnt signaling and potentiates their self-renewal [56]. However, it has been found to be associated with long progression-free survival in patients with solid tumors treated with anti-PD-1/anti-PD-L1 therapies [57]. In a similar manner, transforming growth factor beta (TGF-β) [58], tumor necrosis factor (TNF-α) [59] or paradoxical genes [60] have opposite roles in early tumorigenesis and in later stages of established malignancies.

The main limitation of the study relates to the different characteristics of the two populations being compared: colorectal cancer patients and control subjects. However, the logistic regression studies that have been carried out, considering the variables of age, sex, and BMI (with significant differences between the two populations), allow us to establish the validity of the differences. Additionally, information regarding potential confounding factors, including medication use (with the exception of the lack of antibiotic treatment considered within the exclusion criteria), dietary habits and lifestyle characteristics, was not available. As these factors may influence gut microbiota composition and SCFA levels, residual confounding cannot be excluded. Additionally, the predicted pathways and associations identified in this study were based on bioinformatic inference and were not experimentally validated. Therefore, the lack of functional assays and mechanistic investigations represents an important limitation, as these approaches would be necessary to confirm the biological relevance of the observed associations and to further elucidate the underlying mechanisms linking SCFAs to CRC. Finally, the results obtained in this study must be regarded as preliminary, as the population’s size is modest and the validation in larger independent cohorts is still lacking. If validated, these SCFAs would constitute useful non-invasive biomarkers to complement the methods currently used in clinical practice and improve CRC detection.

## 5. Conclusions

We found a significant association between specific serum SCFA profiles and CRC. These profiles showed reduced levels of valerate and butyrate and increased levels of BSCFAs. Within CRC patients, the highest levels of BSCFAs, particularly isobutyrate and isovalerate, were related to a less invasive cancer. These findings highlight the potential of SCFAs as biomarkers in clinical practice and underscore the need for further investigation in larger cohorts to fully validate their application.

## Figures and Tables

**Table 1:** Clinicopathological variables of the colorectal cancer (CRC) and control subjects.

Variable	CRC Group (N = 36)	Control Group (N = 20)	*p* Value
**Age, median in years (IQR)**	75.50 (67.50–78.50)	58.00 (41.50–64.00)	**<0.001 ^†^**
**Gender, N (%)**			
Male	22 (61.1)	6 (30.0)	**0.026 ^‡^**
Female	14 (38.9)	14 (70.0)	
**BMI group, N (%)**			
Normal weight (BMI < 25 kg/m^2^)	7 (19.4)	3 (15.0)	**0.003 ^‡^**
Overweight (BMI ≥ 25 kg/m^2^ and <30 kg/m^2^)	20 (55.6)	3 (15.0)	
Obese (BMI ≥ 30 kg/m^2^)	9 (25.0)	14 (70.0)	
**Comorbidities, N (%)**			
Dyslipidemia	9 (25.0)	6 (30.0)	0.686 ^‡^
Diabetes	11 (30.6)	3 (15.0)	0.198 ^‡^
AHT	22 (61.1)	7 (35.0)	0.061 ^‡^
**Primary tumor location, N (%)**			
Right colon	18 (50.0)		
Left colon	13 (34.2)		
Rectum	5 (13.2)		
**TNM stage, N (%)**			
I	5 (13.9)		
IIA	9 (25.0)		
IIB	2 (5.6)		
IIC	2 (5.6)		
IIIA	1 (2.8)		
IIIB	13 (36.1)		
IIIC	2 (5.6)		
IV	2 (5.6)		
**T descriptor, N (%)**			
T1	2 (5.6)		
T2	4 (11.1)		
T3	22 (61.1)		
T4	8 (22.2)		
**N descriptor, N (%)**			
N0	19 (52.8)		
N1	13 (36.1)		
N2	4 (11.1)		
**M descriptor, N (%)**			
M0	34 (94.4)		
M1	2 (5.6)		

BMI: body mass index; IQR: interquartile range; AHT: arterial hypertension; TNM: tumor–node–metastasis; N descriptor: lymph node invasion; M descriptor: metastasis; T descriptor: tumor size; ^†^ Mann–Whitney U test; ^‡^ Chi-squared test.

**Table 2:** Mass spectrometry (MS) parameters for each dansylhydrazine-derivatized short-chain fatty acid (SCFA), measured in positive polarity.

Compound	MS/MS Parameters					RT (Min)
	Precursor Ion (*m*/*z*)	Product Ion (*m*/*z*)	Frag (V)	CE (V)	Cell Acc (V)	
**Acetic acid-1-^13^C (IS)**	309.1	171	119	25	4	2.9
**Acetic acid**	308.1	171	119	25	4	2.9
**Propionic acid-1-^13^C (IS)**	323.1	171	129	25	4	4.8
**Propionic acid**	322.1	171	129	25	4	4.8
**Butyric acid-1-^13^C (IS)**	337.1	171	124	29	4	6.9
**Isobutyric acid**	336.1	171	124	25	4	6.6
**Butyric acid**	336.1	171	124	29	4	6.9
**2-Methylbutyric acid**	350.1	171	129	25	4	8.7
**Isovaleric acid**	350.1	171	129	25	4	9.1
**Valeric acid**	350.1	171	129	25	4	9.3

IS: internal standard; *m*/*z*: mass-to-charge ratio; Frag (V): fragmentation voltage; CE (V): collision energy (volts); Cell Acc: cell accelerator voltage; RT: retention time.

**Table 3:** Comparison of serum **SCFA** concentrations between CRC patients and control subjects.

SCFA	Median Serum Concentration in µM (IQR)		*p* Value (U Test)
	CRC Group (N = 36)	Control Group (N = 20)	
**Total SCFAs**	132.85 (86.53–211.95)	56.89 (39.15–160.86)	0.010
**Acetate**	115.49 (81.37–205.77)	58.15 (32.12–150.22)	0.010
**Propionate**	1.86 (0.90–3.05)	1.30 (1.01–1.96)	0.166
**Butyrate**	0.80 (0.51–8.47)	2.72 (2.11–3.70)	0.017
**Valerate**	0.21 (0.06–0.30)	0.43 (0.33–0.67)	<0.001
**BSCFAs**	2.40 (1.84–3.07)	1.62 (1.24–2.15)	0.008
**Isobutyrate**	0.94 (0.72–1.47)	0.73 (0.42–0.93)	0.034
**Isovalerate**	0.78 (0.46–1.17)	0.43 (0.11–0.92)	0.026
**2-Methylbutyrate**	0.55 (0.43–0.66)	0.39 (0.31–0.54)	0.022

µM: micromolar; U test: Mann–Whitney U test; Total SCFAs: sum of acetate, propionate, butyrate, valerate, isobutyrate, isovalerate and 2-methylbutyrate; BSCFAs: total branched short-chain fatty acids (sum of isobutyrate, isovalerate and 2-methylbutyrate).

**Table 4:** Sensitivity analyses evaluating the association and discriminative performance of SCFAs as biomarkers for CRC diagnosis.

SCFA	Firth Logistic Regression		Bootstrapping		K Cross-Validation		
	OR (95% IC) ^†^	*p* Value	AUC After Bootstrapping ^‡^	Optimism	% Iterations with *p* < 0.05	K-Cross-Validated AUC	Optimism
**Total SCFAs**	3.62 (0.80–16.48)	0.096	0.732	0.02	40%	0.462	0.25
**Acetate**	2.72 (0.60–12.33)	0.195	0.708	<0.00	0.0%	0.475	0.23
**Propionate**	1.25 (0.32–4.94)	0.746	0.613	<0.00	0.0%	0.568	0.05
**Butyrate**	0.07 (0.01–0.43)	0.004	0.695	0.01	100%	0.543	0.15
**Valerate**	0.24 (0.06–1.02)	0.054	0.813	<0.00	30%	0.787	0.03
**BSCFAs**	8.86 (1.63–48.07)	0.012	0.717	<0.00	100%	0.679	0.04
**Isobutyrate**	12.9 (1.69–98.14)	0.014	0.672	<0.00	100%	0.622	0.05
**Isovalerate**	3.92 (0.85–18.12)	0.080	0.681	<0.00	30%	0.472	0.21
**2-Methylbutyrate**	8.73 (1.80–42.4)	0.007	0.686	<0.00	100%	0.668	0.02

^†^ For logistic regression calculations, categorized SCFA concentrations were used. ^‡^ For AUC calculation, SCFA concentrations were used as continuous variables.

**Table 5:** Comparison of serum SCFA concentrations between CRC cases with different TNM stage.

SCFA	Median Serum Concentration in µM (IQR)		*p* Value (U Test)
	I–IIIA Group (N = 19)	IIIB–IV Group (N = 17)	
**Total SCFAs**	157.22 (110.43–289.38)	99.27 (72.75–147.42)	0.028
**Acetate**	146.09 (95.89–283.10)	93.74 (61.84–140.17)	0.041
**Propionate**	2.58 (1.84–3.14)	1.15 (0.55–1.73)	0.028
**Butyrate**	1.11 (0.58–11.10)	0.63 (0.50–0.86)	0.051
**Valerate**	0.23 (0.09–0.35)	0.13 (0.05–0.29)	0.222
**BSCFAs**	2.60 (2.01–3.13)	2.00 (1.54–2.78)	0.103
**Isobutyrate**	1.09 (0.8–1.61)	0.84 (0.64–0.98)	0.084
**Isovalerate**	0.84 (0.72–1.22)	0.56 (0.36–0.88)	0.090
**2-Methylbutyrate**	0.56 (0.45–0.68)	0.52 (0.43–0.61)	0.623

Total SCFAs: sum of acetate, propionate, butyrate, valerate, isobutyrate, isovalerate and 2-methylbutyrate; BSCFAs: sum of isobutyrate, isovalerate and 2-methylbutyrate.

**Table 6:** Comparison of serum SCFA concentrations between CRC cases with different degrees of lymph node invasion (N descriptor).

SCFA	Median Serum Concentration in µM (IQR)		*p* Value (U Test)
	N0 Group (N = 19)	N1/N2 Group (N = 17)	
**Total SCFAs**	157.22 (114.79–289.38)	99.27 (72.76–147.42)	0.023
**Acetate**	146.09 (103.56–283.10)	93.74 (61.84–140.17)	0.028
**Propionate**	2.23 (1.36–2.90)	1.28 (0.90–3.05)	0.274
**Butyrate**	1.09 (0.51–9.91)	0.63 (0.55–1.18)	0.350
**Valerate**	0.24 (0.09–0.35)	0.13 (0.05–0.29)	0.188
**BSCFAs**	2.43 (1.90–3.07)	2.39 (1.68–2.82)	0.456
**Isobutyrate**	1.00 (0.75–1.45)	0.87 (0.68–1.38)	0.496
**Isovalerate**	0.81 (0.62–1.22)	0.62 (0.39–1.01)	0.318
**2-Methylbutyrate**	0.55 (0.45–0.68)	0.53 (0.43–0.61)	0.812

Total SCFAs: sum of acetate, propionate, butyrate, valerate, isobutyrate, isovalerate and 2-methylbutyrate; BSCFAs: sum of isobutyrate, isovalerate and 2-methylbutyrate.

## Data Availability

The raw data sequences related to bacterial taxonomy in feces and serum can be found in online repositories: Submission ID: SUB14179719, https://submit.ncbi.nlm.nih.gov/subs/sra/SUB14179719, accessed on 18 March 2024; and http://www.ncbi.nlm.nih.gov/bioproject/1211866, BioProject ID: PRJNA1211866, accessed on 27 January 2025.

## Supplementary Materials

The following supporting information can be downloaded at https://www.mdpi.com/article/10.3390/cells15121096/s1, Table S1: List of SCFA-producing bacterial genera considered for the study and the reference supporting their characterization as SCFA-producers. Table S2: Metacyc pathways predicted by PICRUSt2 that are involved in SCFA production and degradation considered in this study. Pathways producing byproducts that feed into these routes were not included in the analysis.
