# Supplementary material for: Serum Short-Chain Fatty Acids in Colorectal Cancer: Diagnostic Performance and Decoupling from Gut Producer Abundance
Source: Cells. 2026 Jun 16;15(12):1096. doi: 10.3390/cells15121096 (PMC13297175; doi:10.3390/cells15121096)
Supplement: Supplementary file 1 [file cells-15-01096-s001.zip › cells-4363259-supplementary.pdf]

Supplementary material. Serum short-chain fatty acids as diagnostic biomarkers in surgically resected CRC patients and their relationship with clinicopathological variables

Table S1. List of SCFA-producing bacterial genera considered for the study and the reference supporting their characterization as SCFA-producers.

**Genera**	**Reference**	**Genera**	**Reference**
Acetobacter	[24]	*Eubacterium_eligens_group*	[29]
Acetobacterium	[25]	*Eubacterium_fissicatena_group*	[29]
Akkermansia	[26]	*Eubacterium_hallii_group*	[29]
Anaerostipes	[27]	*Eubacterium_oxidoreducens_group*	[29]
Anaerotruncus	[28]	*Eubacterium_ruminantium_group*	[29]
Bacteroides	[29]	*Eubacterium_ventriosum_group*	[29]
Bifidobacterium	[29]	*Eubacterium_xylanophilum_group*	[29]
Blautia	[29]	*Faecalibacterium*	[29]
Butyricicoccus	[27]	*Intestinibacter*	[32]
Butyricimonas	[27]	*Lachnoclostridium*	[29]
Butyrivibrio	[27]	*Lachnospira*	[33]
Clostridium_sensu_stricto_1	[29]	*Lactobacillus*	[29]
Clostridium_sensu_stricto_10	[29]	*Megasphaera*	[31]
Clostridium_sensu_stricto_12	[29]	*Oscillibacter*	[34]
Clostridium_sensu_stricto_13	[29]	*Parabacteroides*	[29]
Clostridium_sensu_stricto_18	[29]	*Phascolarctobacterium*	[31]
Clostridium_sensu_stricto_2	[29]	*Prevotella*	[26,29]
Clostridium_sensu_stricto_3	[29]	*Propionibacterium*	[35]
Clostridium_sensu_stricto_4	[29]	*Pseudobutyrivibrio*	[27]
Clostridium_sensu_stricto_5	[29]	*Romboutsia*	[36]
Clostridium_sensu_stricto_6	[29]	*Roseburia*	[29]
Clostridium_sensu_stricto_8	[29]	*Ruminiclostridium*	[29]
Clostridium_sensu_stricto_9	[29]	*Ruminococcus*	[29]
Coprococcus	[29]	*Ruminococcus_gauvreauii_group*	[29]
Desulfovibrio	[30]	*Ruminococcus_gnavus_group*	[29]
Dialister	[31]	*Ruminococcus_torques_group*	[29]
Dorea	[29]	*Subdoligranulum*	[27]
Erysipelatoclostridium	[29]	*Veillonella*	[29]
Eubacterium	[29]		
Main SCFA-producing bacterial families: f__Lachnospiraceae and f__Ruminococcaceae			[37]
Main SCFA-producing bacterial phyla: p__Firmicutes and p__Bacteroidota			[37]

Table S2. Metacyc pathways predicted by PICRUSt2 that are involved in SCFA production and degradation considered in this study. Pathways producing by-products that feed into these routes were not included in the analysis.

SCFAs	Metacyc
Acetate	- Bifidobacterium shunt<br>- Hexitol fermentation to lactate, formate, ethanol and acetate<br>- Pyruvate fermentation to acetate and lactate II<br>- Mixed acid fermentation<br>- L-lysine fermentation to acetate and butanoate<br>- Superpathway of Clostridium acetobutylicum acidogenic fermentation
Propionate	- L-glutamate degradation VIII (to propanoate)<br>- Pyruvate fermentation to propanoate I<br>- 2-methylcitrate cycle I<br>- 2-methylcitrate cycle II
Butanoate	- Acetyl CoA fermentation to butanoate II<br>- Pyruvate fermentation to butanoate<br>- L-lysine fermentation to acetate and butanoate<br>- Succinate fermentation to butanoate<br>- Superpathway of Clostridium acetobutylicum acidogenic fermentation
Valerate	None of the routes could be analyzed as being involved in valerate production and degradation directly
Isobutyrate	- L-valine degradation I
Isovalerate	- L-leucine degradation I
2-methylbutyrate	- L-isoleucine degradation I
